# A Beacon Transmission Power Control Algorithm Based on Wireless Channel Load Forecasting in VANETs

**DOI:** 10.1371/journal.pone.0142775

**Published:** 2015-11-16

**Authors:** Yuanfu Mo, Dexin Yu, Jun Song, Kun Zheng, Yajuan Guo

**Affiliations:** 1 College of Transportation, Jilin University, Changchun, China; 2 Dalian International Airport, Dalian, China; Tianjin University of Technology, CHINA

## Abstract

In a vehicular ad hoc network (VANET), the periodic exchange of single-hop status information broadcasts (beacon frames) produces channel loading, which causes channel congestion and induces information conflict problems. To guarantee fairness in beacon transmissions from each node and maximum network connectivity, adjustment of the beacon transmission power is an effective method for reducing and preventing channel congestion. In this study, the primary factors that influence wireless channel loading are selected to construct the KF-BCLF, which is a channel load forecasting algorithm based on a recursive Kalman filter and employs multiple regression equation. By pre-adjusting the transmission power based on the forecasted channel load, the channel load was kept within a predefined range; therefore, channel congestion was prevented. Based on this method, the CLF-BTPC, which is a transmission power control algorithm, is proposed. To verify KF-BCLF algorithm, a traffic survey method that involved the collection of floating car data along a major traffic road in Changchun City is employed. By comparing this forecast with the measured channel loads, the proposed KF-BCLF algorithm was proven to be effective. In addition, the CLF-BTPC algorithm is verified by simulating a section of eight-lane highway and a signal-controlled urban intersection. The results of the two verification process indicate that this distributed CLF-BTPC algorithm can effectively control channel load, prevent channel congestion, and enhance the stability and robustness of wireless beacon transmission in a vehicular network.

## Introduction

In an “active safety” system of a vehicular ad hoc network (VANET), intelligent vehicles cooperate to avoid dangerous situations and traffic accidents. Vehicles (in the subsequent text, “vehicle” and “node” refer to the same concept in a VANET) establish mutual perceptions by periodically exchanging single-hop status information broadcasts, which include geographical location, speed, and driving direction [1, 2]. Mutual perception between vehicles can be employed to detect dangerous traffic conditions, such as traffic jams and passing vehicles. This type of time-varying periodic status information exchange is referred to as beacon frames [3–5]. In an environment with a high communication density, the prevention of wireless communication performance degradation due to the large number of vehicle-generated beacon messages, i.e., congestion control is a serious challenge of VANETs that requires resolution [6, 7].

According to existing studies, many researchers believe that the design of a wireless channel load control strategy for a VANET is needed [8–10]. The beacon in a VANET can be categorized into two types: a periodic beacon and an event-driven beacon [11, 12]. A periodic beacon is a basic component of a VANET [13]. According to studies by Habib [14] and Javadi [15], periodic beacon messages may cause channel saturation and congestion. The only congestion control measure that was proposed in the draft of the Institute of Electrical and Electronics Engineers (IEEE) standard 802.11p is as follows: when channel transmission occupancy rate of greater than 50% is detected, all messages are blocked, with the exception of messages with maximum priority [16–18]. However, this measure will not resolve channel congestion caused by periodic beacon messages.

How do we control the channel load produced by periodic beacon messages? Essentially, two methods can be employed: adjustment of the beacon transmission power or adjustment of the beacon generation rate [19–22]. In the safe application of a VANET, high beacon message generation rate can increase information accuracy. In addition, information from high-density periodic beacon messages is required to detect potential danger. Therefore, a beacon message cannot be simply discarded, delayed, or reduced; conversely, the channel load carried by the periodic beacon messages should be controlled by adjusting the transmission power. Increase or decrease in the transmission power changes the number of vehicles within the communication range that compete for channel space and may change the channel load. Mean [23] adjusted the transmission power to create a highly connected vehicle network. Giuseppe [24] proposed a time division multiple access (TDMA) reservation mechanism to control vehicle’s transmission power by forcing the number of surrounding vehicles to remain within a pre-defined range and maintaining the channel load within a certain range of values. Khorakhum [25] proposed transmission power adjustments based on necessary time restrictions for busy channels within a network range. When a vehicle required additional transmission power, its transmission power was assessed to determine whether it was less than the average transmission power; if the vehicle’s transmission power exceeded the average transmission power, the power increase was delayed.

These methods involve the adjustment of a vehicle’s transmission power based on the traffic flow density (vehicle density) or the channel congestion when controlling channel loading. Although these methods enable vehicles to react to changes of channel conditions, they cannot help vehicles to avoid channel congestion. If a vehicle’s channel load can be effectively predicted, a vehicle can adjust its transmission power in advance based on the estimated load. Channel congestion can be avoided in the fundamental. So the communication performance of a VANET can be optimized. This type of pre-estimation-based congestion control mechanism has not been investigated in VANETs.

This paper proposes a beacon transmission power control algorithm based on channel load forecasting in VANETs; it encompasses the following steps: (1) select the most influential factors in determining wireless channel load, construct a regression model and then perform Kalman-filter-based channel load forecasting, and (2) predefine maximum and minimum thresholds for a channel, determine the beacon transmission power after comparing the forecasted channel load with the predefined threshold values, and establish a beacon transmission power control algorithm based on “channel load forecasting and comparison”.

The remainder of this paper is organized as follows: In Section 2, we propose the KF-BCLF algorithm. In Section 3, we present an example of the KF-BCLF algorithm. In Section 4, we propose the CLF-BTPC algorithm. Section 5 verifies the CLF-BTPC algorithm by simulations. We present our conclusions in Section 6.

## KF-BCLF Algorithm

The Federal Communications Commission (FCC) has allocated 75-MHz band at 5.9 GHz for VANETs. This band is divided into seven channels with 10-MHz width for each channel. One channel is reserved for safety-related information exchanges, whereas the remaining six channels are employed for non-safety-related applications (FCC 2004). IEEE 802.11p provides a data transmission speed range of 3–27 Mbps for the 10-MHz channel (2008).

In a VANET, the communication process for traveling vehicles forms a large-scale nonlinear system [26, 27]; it is influenced by random factors, such as vehicle operating property, vehicle performance, traffic flow conditions, communication parameters, and the communication environment [28, 29]. Periodic status information (source of beacon massages) produces channel loads. From the standpoint of the channel load surrounding each vehicle, this load is not only influenced by the expected periodical information generation rate, average message size, configured transmission power, and channel fading conditions but is also related to factors such as traffic flow, traffic density, and road incidents. Kalman filter theory employs a small number of parameters and is computationally convenient [30, 31]. However, the conventional type of model that utilizes Kalman filtering is based on historical channel load data and does not consider many influential factors in a subsequent time period, which causes shortcomings in forecasting accuracy and self-adaptability. To improve the channel load forecasting accuracy, a multivariate relationship model must be constructed to evaluate the channel load based on the selection of possible influential factors.

This study combines the characteristics of Kalman filter, selects *m* factors that influence the channel load [32], and establishes a multiple regression-based KF-BCLF algorithm. The specific steps are as follows:

Step 1: Construct multiple linear regression equations that reflect the relationship between the channel load and the influential factors:
  $$\left\{ \begin{array}{l} x_{0}(k+1)=b_{00}x_{0}(k)+b_{01}x_{1}(k)+b_{02}x_{2}(k)+\cdots +b_{0m}x_{m}(k)+\varepsilon_{0}\\ x_{1}(k+1)=b_{10}x_{0}(k)+b_{11}x_{1}(k)+b_{12}x_{2}(k)+\cdots +b_{1m}x_{m}(k)+\varepsilon_{1}\\ \;\vdots \\ x_{m}(k+1)=b_{m0}x_{0}(k)+b_{m1}x_{1}(k)+b_{m2}x_{2}(k)+\cdots +b_{mm}x_{m}(k)+\varepsilon_{m} \end{array}\right.$$ (1)
  In Eq (1), *b*_*00*_, *b*_*01*_, *…*,*b*_*mm*_ and *ε*_*0*_, *ε*_*1*_, *…*, *ε*_*m*_ are regression coefficients that can be obtained using the method of least squares.
  $$\left[\begin{array}{cccc} \varepsilon_{0} & \varepsilon_{1} & \cdots & \varepsilon_{m}\\ b_{00} & b_{10} & \cdots & b_{m0}\\ b_{01} & b_{11} & \cdots & b_{m1}\\ \vdots & \vdots & & \vdots \\ b_{0m} & b_{1m} & \cdots & b_{mm} \end{array}\right]=\;{\left[T^{\prime }•T\right]}^{-1}T^{\prime }•\left[\begin{array}{ccccc} x_{0}(2) & x_{1}(2) & x_{2}(2) & \cdots & x_{m}(2)\\ x_{0}(3) & x_{1}(3) & x_{2}(3) & \cdots & x_{m}(3)\\ \vdots & \vdots & \vdots & & \vdots \\ x_{0}(n) & x_{1}(n) & x_{2}(n) & \cdots & x_{m}(n) \end{array}\right]$$ (2)
  In Eq (2), $T=\left[\begin{array}{ccccc} 1 & x_{0}(1) & x_{1}(1) & \cdots & x_{m}(1)\\ 1 & x_{0}(2) & x_{0}(2) & \cdots & x_{m}(2)\\ \vdots & \vdots & \vdots & & \vdots \\ 1 & x_{0}(n-1) & x_{1}(n-1) & \cdots & x_{m}(n-1) \end{array}\right].$
Step 2: From Eq (1), construct the state equation as follows:
  Let *X*(*k*) = [*x*_0_(*k*) *x*_1_(*k*)…*x*_m_(*k*)]^*'*^, then:
  $$\left\{ \begin{array}{l} X(k+1)=B(k)X(k)+w(k)\\ y(k)=A(k){\left[x_{0}(k+1)\;x_{1}(k+1)\;\cdots \;x_{m}(k+1)\right]}^{\prime }+V(k) \end{array}\right.$$ (3)
  In Eq (3), $B(k)=\left[\begin{array}{cccc} b_{00} & b_{01} & \cdots & b_{0n}\\ b_{10} & b_{11} & \cdots & b_{1n}\\ \vdots & \vdots & & \vdots \\ b_{m0} & b_{m1} & \cdots & b_{mn} \end{array}\right]$; *A*(*k*) = [1 0…0]; *w*(*k*) = [*ε*_0_ *ε*_1_…*ε*_*m*_]*'*;
  and *V*(*k*) is the measured noise at time *k*.
Step 3: Initialize the filter variance matrix *P*(*0*) and the measured value *X*(*0*).
Step 4: Recursively calculate the filter coefficients:
  $$P(k|k-1)=B(k)P(k-1)B^{\prime }(k)+Q(k-1)$$ (4)
  In Eq (4), *Q*(*k*-1) is a nonnegative definite matrix;
  $$K(k)=P(k|k-1)A^{\prime }(k){\left[A(k)P(k|k-1)A^{\prime }(k)+R(k)\right]}^{-1}$$ (5)
Step 5: Update the status using
  $$\overset{⌢}{x}(k)=B(k)\overset{⌢}{x}(k-1)+K(k)\left[y(k)-A(k)B(k)\overset{⌢}{x}(k-1)\right]$$ (6)
  In Eq (6), *y*(*k*) = *x*_0_(*k*);
  P(k)=[I−K(k)A(k)]P(k|k−1)  .  (7)
Step 6: Let *k* = *k*+1; return to Step 4 and repeat the computation until the termination condition is satisfied.
Step 7: Calculate the channel load forecast value using *y*(*k*) = *A*(*k*)•*X*(*k*).

## Example of the KF-BCLF algorithm

To support active vehicle safety applications and to increase the accuracy required for safety applications, several location messages must be generated every second by a vehicle. In extremely urgent safety applications, such as collaborative front collision warning systems, the periodic rate can exceed 10–20 messages per second [33, 34]. In a safety study, Maxim [35] noted that the size of a periodic status message ranges from 250 to 800 bytes for current digital signatures and certificates.

To verify the effectiveness of the KF-BCLF algorithm proposed in this paper, a traffic survey method was employed. The floating car was employed to collect data on a segment of Renmin Street (between Jiefang Road and Nanhu Road) in Changchun City. Once every 5 minutes, the floating car collected traffic data on the speed, number of vehicles within communication range, and traffic flow density around it. The data comprised 300 points; the last 24 points were employed to verify front sampling data. Using the channel load (*CL*) sequence {*x*(*k*)}(*k* = 1,2,…,300) as the subject of study, a short-term *CL* forecasting experiment was conducted by using KF-BCLF algorithm.

For computational convenience, the transmission power of each vehicle was assumed to be 10 dBm, the communication range was set to 500 m, the size of the periodic status messages size was 800 bytes, and the periodic messages were generated at a rate of 20 per second. The *CL* near the floating car was computed using the following equation, the result was applied as the measured *CL*.

$$Measured\_CL=\frac{cars}{com\_dis\text{tan}ce}*20\left[pkts/s\right]*800\left[B/pkt\right]*8\left[bits/B\right]$$ (8)

In Eq (8), *Measured_CL* represents the measured *CL* and $\frac{cars}{com\_dis\text{tan}ce}$ is the number of vehicles within the communication range. To simplify the *CL* forecasting computation, this study selected the traffic density and floating car speed as the variable parameters in the *CL* regression equation to construct the *CL* forecasting model. The values of the regression parameters were determined using the method of least squares. The single-step *CL* forecasting value was obtained according to Kalman recursion model and the measured *CL*. To compare the results, a relative error indicator was introduced as follows:

$$rerr=\frac{L_{pred}(t)-L_{real}(t)}{L_{real}(t)}$$ (9)

**Fig 1:**
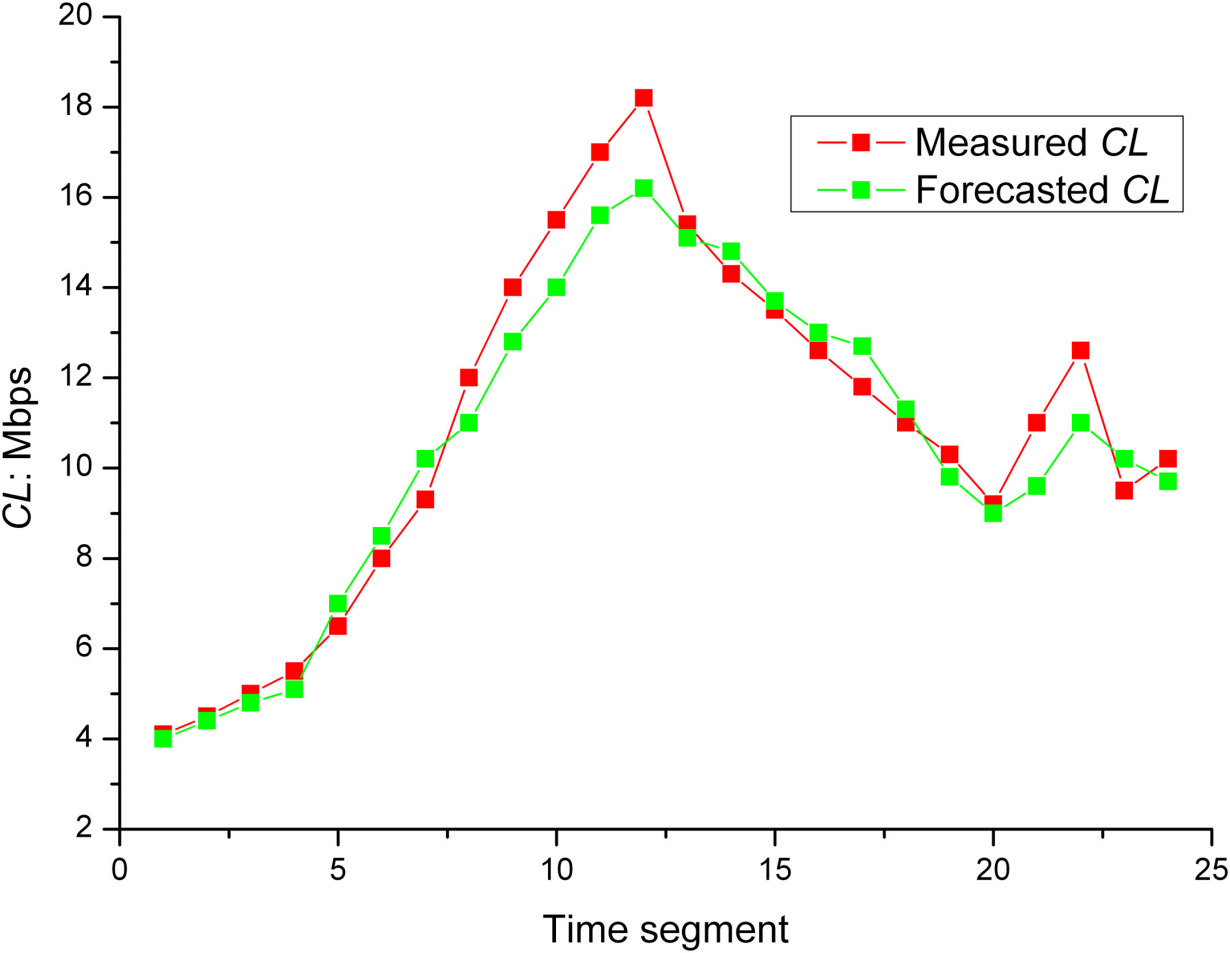
Results of the *CL* forecasting.

**Fig 2:**
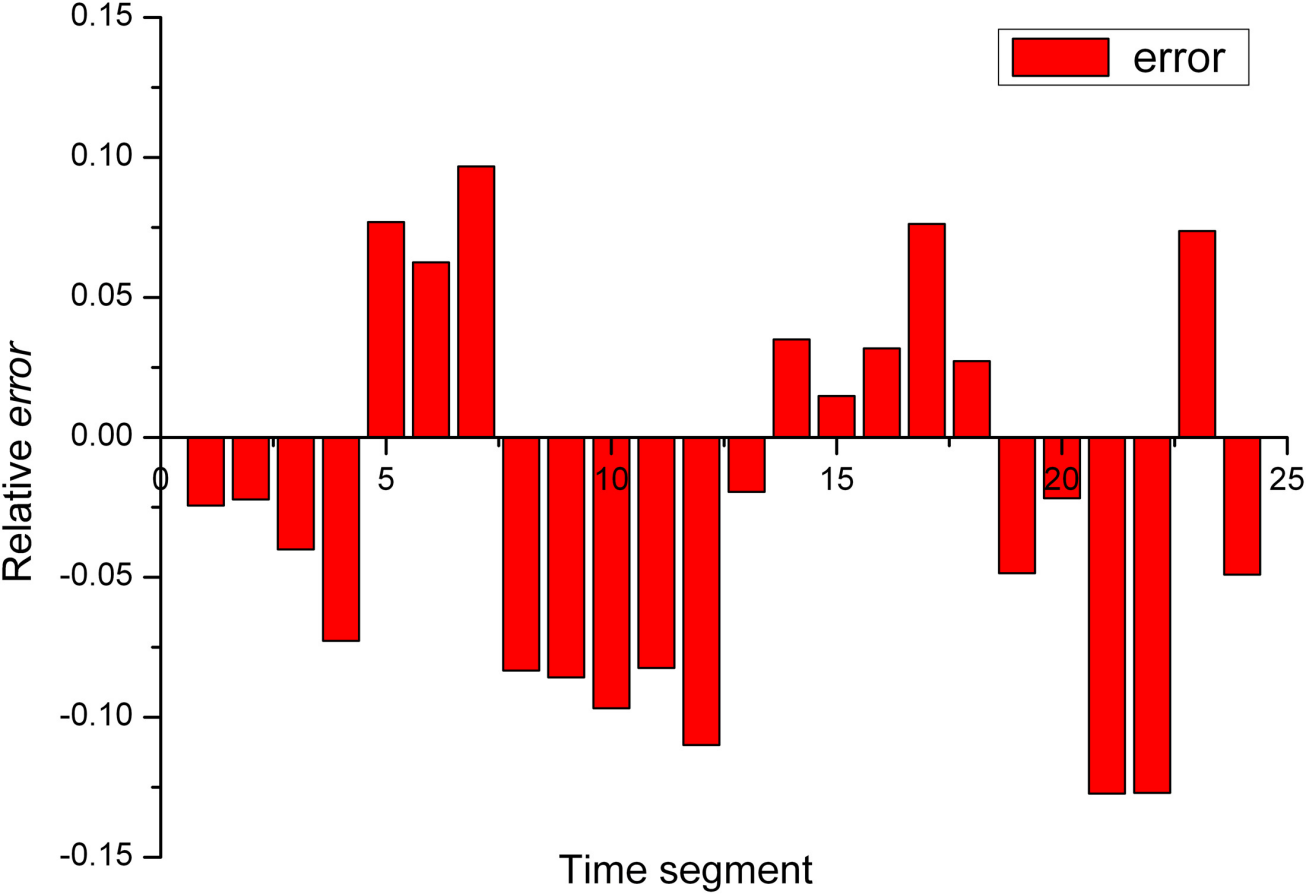
Relative error of the results.

In Eq (9), *rerr* is the relative error, *L*_*pred*_ (*t*) is the *CL* forecasting value, and *L*_*real*_(*t*) is the measured *CL* value. The results are shown in the following graph:

A shown in Figs 1 and 2, KF-BCLF algorithm considered the factors that exhibited the greatest influence on the *CL*. The maximum forecasting error was 13% The forecasting accuracy was satisfactory, which indicates that this algorithm is effective for *CL* forecasting.

## CLF-BTPC algorithm

In mobile network transmission power control algorithm, the goals for overall system capacity maximization, energy consumption, or point-to-point communication connectivity are typically established [36–38]. Because the inter-vehicular communication mode in a VANET environment is generally one-to-many [39–41], energy consumption is generally not an issue; therefore, existing studies are not applicable for a VANET.

Using higher transmission power may produce longer transmission distance and increase the robustness of message transmission but will cause channel saturation and a greater message conflict ratio [42, 43]. In a safety application, fairness in the communication must be considered [44, 45]. Because a VANET does not have a central communications coordinator, a distributed algorithm must be employed to optimize the beacon message transmission power and ensure fairness among all nodes; otherwise, dangerous conditions may be created for surrounding nodes [46, 47]. When designing and optimizing an algorithm to control transmission power, the transmission power of each node within the communication range must be fairly assigned in a way that satisfies the safety requirements for the purpose of each individual node increasing their total packet transmission efficiency.

We consider a scenario in which a set of vehicles (also referred to as nodes) is moving along a road. The nodes periodically send beacon messages to inform the nodes in the vicinity of their current position, direction, and velocity. We assume that the beaconing frequency is identical for all nodes. However, the power for transmitting beacon messages can be adjusted to control the channel load. A short version of the statement and definitions, which is required to prove Theorem 1, is presented as follows:

**Assumption 1:** A group of nodes *N* = {*u*_1_,…,*u*_*n*_} moves in a straight line with length *R* = [0,1] (for simplicity, the road is considered to be a straight line); for *u*_*i*_∈*N*, where *x*(*i*,*t*) represents the location of node *u*_*i*_ at time *t* with *x*∈[0,1].
**Assumption 2**: Node *u*_*i*_∈*N* periodically transmits beacon messages to other nodes *u*_*j*_∈*N*, *j≠i* with the constant frequency *f*. The initial beacon transmission power is *P*_*ini*_∈[0,*P*_max_], where *p*_*max*_ is the maximum allowable transmission power.

The following terms can be defined:

**Definition 1:** *Power assignment* (*PA*): Given a set of nodes *N* = {*u*_1_,…,*u*_*n*_}, for ∀*u*_*i*_∈*N*, ∃*PA*(*i*), let node *u*_*i*_ transmit beacon messages at a transmission power of *PA*(*i*)·*p*_*ini*_∈[0,*p*_max_].
**Definition 2:** *Carrier sensing range* (*CSR*): For ∀*u*_*i*_∈*N*, given the power assignment *PA*(*i*), a unique *CSR*, which is denoted by *CSR*(*PA*,*i*), always exists.
**Definition 3:** *Channel load* (*CL*): For ∀*u*_*i*_∈*N*, given a power assignment *PA*(*i*), the wireless *CL* surrounding node *u*_*i*_ is *CL*(*PA*,*i*) = |{*u*_*j*_∈*N*, *j≠i*: *u*_*j*_∈*CSR*(*PA*,*i*)}|.
**Definition 4**: Maximum and minimum *CL* (max_*CL*(*PA*) and min_*CL*(*PA*)): For ∀*u*_*i*_∈*N*, at a location *x*, *u*_*j*_∈*CSR*(*PA*,*i*),*j*≠*i* exists, i.e., if *u*_*j*_ is a node within the *CSR* of *u*_*i*_, then the maximum *CL* for all nodes within the *CSR* of node *u*_*i*_ is $\text{max}\_CL(PA)=\underset{x\in \left[0,1\right]}{\text{max}}CL(PA,x)$and the minimum *CL* for all nodes within the *CSR* of node *u*_*i*_ is $\text{min}\_CL(PA)=\underset{x\in \left[0,1\right]}{\text{min}}CL(PA,x)$
**Definition 5:** *CL forecasting* (*CLF*): For ∀*u*_*i*_∈*N*, the *CLF* at time *t* can be obtained from the KF-BCLF algorithm.
**Definition 6:** *CL forecasting-based transmission power assignment problem* (***TPAP***): Given a set of nodes *N* = {*u*_1_,…,*u*_*n*_} in *R* = [0,1], determine the maximum *PA* for each node based on the *CLF* under the premise that the real *CL* of each node is controlled within an allowable range.

If the *PA* of a beacon message is increased, the robustness to power fluctuation and interference also increases, which indicates that the message can be transmitted over a longer distance. If the *PA*s of all nodes in the network simultaneously increases, then the *CSR* and the number of channel-sharing nodes of each vehicle also increases, which decreases the spatial multiplexing rate. According to a study by Torrent [48], under highway conditions, when the beacon communication distance is 300 m, if the transmission power is increased from 10 dBm to 20 dBm, the wireless *CL* of the emitting end increases from 2.58 Mbps to 18.5 Mbps, which reduces the beacon receiving ratio at the receiving end from 0.6 to 0.1. Therefore, when assigning the beacon transmission power, a suitable balance should be achieved to determine the optimal operating strategy.

The objective of this CLF-BTPC algorithm is to fairly assign the *PA* (assigned power) to every vehicle in a distributed manner while satisfying the condition that the *CL* around each vehicle is guaranteed to be within the predefined threshold range. The beacon transmission power of each vehicle is maximized; therefore, the connectivity of the mobile network is also maximized. CLF-BTPC algorithm is as follows:

1. **Input**: *forecast_load*, *max_adjust_load*, *min_adjust_load*, *PA*,*N* = {*u*_1_,…,*u_n_*}, *ε*

2. **Output**: *PA*

3. If (*forecast_load*≤*min_adjust_load*) then

4.  while (*max_CL*(*PA*)≤*max_adjust_load*) do

5.   for (*j* = 1 to *n*,* j≠i*)do

6.    *PA*(*j*) = *PA*(*j*)+*ε*

7.   end for

8.  end while

9.  for (*j* = 1 to *n*,*j≠i*) do

10.   *PA*(*j*) = *PA*(*j*)-*ε*

11.  end for

12. Else

13. If (*forecast_load*≥*max_adjust_load*) then

14.  while (*min_CL*(*PA*)≤*min_adjust_load*) do

15.   for (*j* = 1 to *n*,*j≠i*) do

16.    *PA*(*j*) = *PA*(*j*)-*ε*

17.   end for

18.  end while

19.  for (*j* = 1 to *n*,*j≠i*) do

20.   *PA*(*j*) = *PA*(*j*)+*ε*

21.  end for

22. End if

23. End if

### Algorithm 1 CLF-BTPC algorithm

In Algorithm 1, *forecast_load* is the channel load forecast value, the *max_adjust_load* is the maximum allowable channel load, the *min_adjust_load* is the minimum allowable channel load, *PA* is the beacon’s assigned power, and *ε* is the step size for power adjustments.

**Theorem 1**: The value of the obtained *PA* using the CLF-BTPC algorithm is the optimal solution to the *TPAP*.

Proof: The transmission power that corresponds to the transmission power assignment *PA* from algorithm 1 is *p*_*max*_. If ∃*PA*'>*PA* is the maximum assigned transmission power, then the corresponding transmission power is *P*', and *p*'>*p*_max_. According to Definition 4, *CL*(*PA*',*i*)<max_*CL*(*PA*) ∀*i*∈1,…,*n*, i.e., *p*'*<p*_max_, which conflicts with a known condition. Therefore, *PA* is the maximum transmission power assigned to the node. The *PA* value obtained from CLF-BTPC algorithm is the optimal solution to the *TPAP*. QED.

The workflow of the CLF-BTPC algorithm is as follows: all nodes in the network transmit periodic beacon messages with the initial transmission power *p*_*ini*_, and the target node forecasts the *CL* once in each time interval. If the *CL*-forecasted value of the target node is less than the lower bound of the predefined *CL* range, then all nodes within the *CSR* of this target node increase their transmission power by *kε* (*k* is the number of steps) until max_CL(PA) is less than the upper boundary of the predefined range. If the *CL* of the target node is higher than the upper bound of the predefined range, then all nodes within the *CSR* of this target node decrease their transmission power by *kε* (*k* is the number of steps) until min_CL(PA) is greater than the lower bound of the predefined range.

## Simulation verifications of the CLF-BTPC algorithm

In this study, a basic section of eight-lane highway and a signal-controlled urban intersection were selected to examine the changes of the *CL* produced by periodic beacon messages before and after the CLF-BTPC algorithm was applied in high-density wireless communication environment. Paramics was selected as the traffic simulation tool. In reference to the requirements for the periodic beacon message generation rate and the packet size for mobile safety applications in the literature [48], the beacon message size was set to 800 bytes, and the generation rate was set to 15 messages per second, i.e., beacon message generation rate is 96 Kbps per vehicle. The initial communication range was 250 m, and the initial *CSR* was 500 m. The maximum communication range was 500 m, and the maximum *CSR* was 1000 m. The minimum *CL* threshold was 3 Mbps, the maximum *CL* threshold was 6 Mbps, the power adjustment step size was 0.01, and the *CL* forecasting interval was 1 min.

### Signalized urban intersection

The traffic simulation parameters for a signalized urban intersection are listed in Table 1.

**Table 1:** Parameters for the urban intersection simulation.

Parameter	Value
Approach (exit) length	1 km
Vehicle location sampling interval	5 s
Average traffic flow per lane	1,600 vph
Approach number	4
Exit number	4
Speed limit	50 km/h
Signal cycle	60 s
Split	1/4
Green time	30 s
Green time interval	30 s

According to the traffic simulation parameters in Table 1, the average vehicle headway is 15 m. Near the intersection, the average *CL* produced by periodic beacon messages is

$$\frac{8\left[lanes\right]*500m\left[com\_diameter\right]}{15m\left[between\;\_cars\right]}=267\left[cars/com\_range\right]\;$$

267[cars]*15pkts/s*800B/pkt*8bits/B=25.6Mbps

**Fig 3:**
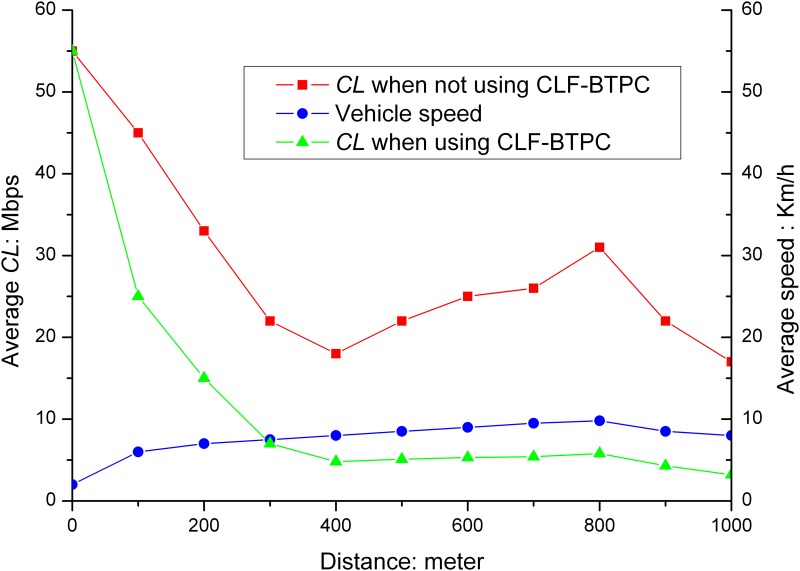
*CL* in exit road section. As shown in Fig 3, after the CLF-BTPC algorithm is applied, the *CL* converged to values within the predefined threshold range of 3–6 Mbps after a period of oscillation in the exit road section. The closer the distances to the exit intersection, the larger the *CL*. When the vehicle moves away from the intersection, the *CL* will significantly decrease. The *CL* begins to increase when the distance from the intersection is 400 m.

**Fig 4:**
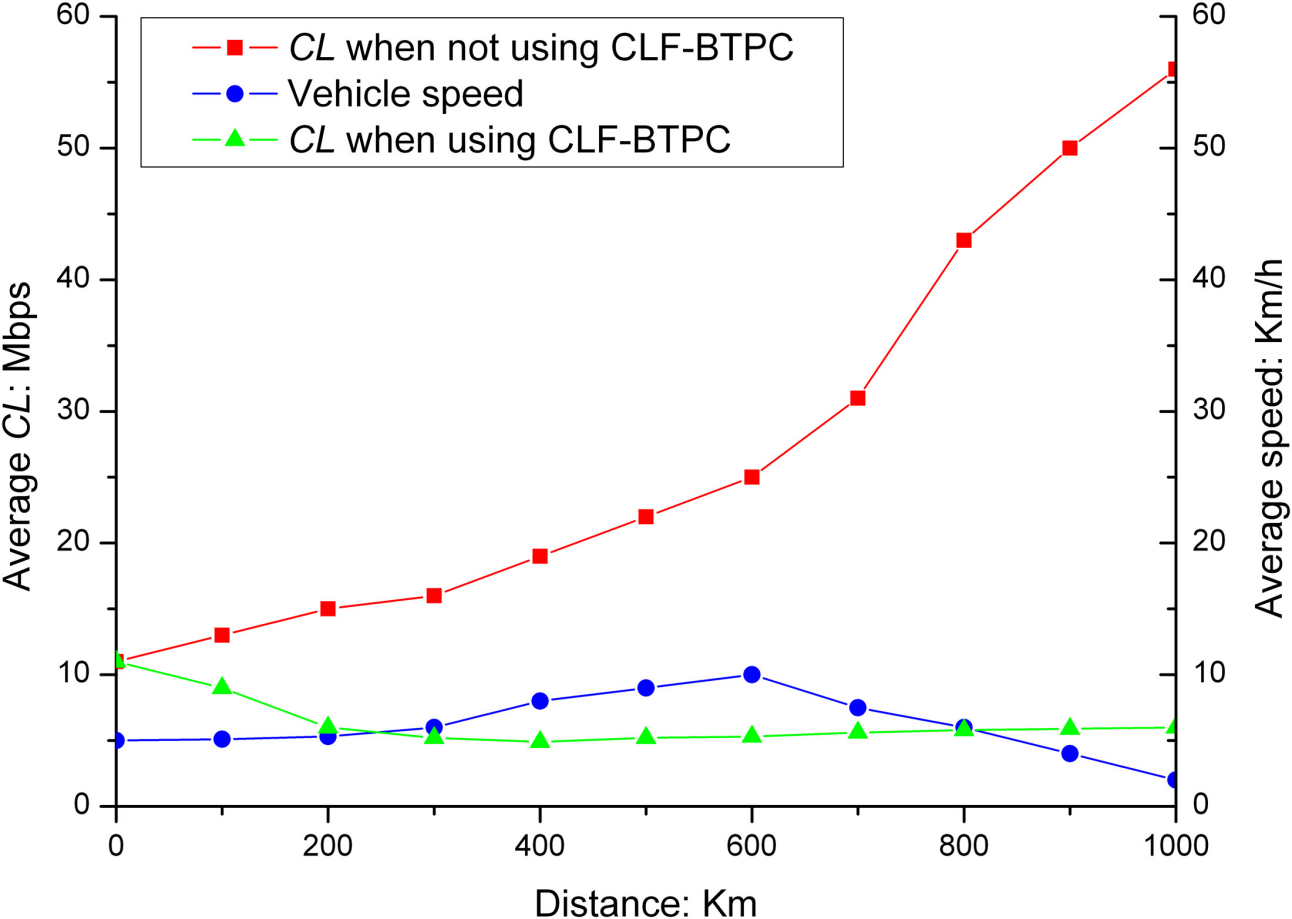
*CL* in the entrance road section. As shown in Fig 4, when vehicles approach the intersection, the *CL* increases with an increase in speed. At 600 m, the maximum vehicle speed is attained. The maximum *CL* is attained near the intersection, and the *CL* value is less than 6 Mbps after the CLF-BTPC algorithm is applied. The results indicate that the CLF-BTPC algorithm is more effective for frequent changes in vehicle speeds (e.g., near the intersection).

When the CLF-BTPC algorithm was applied, the average *CL*s on the exit and entrance road sections are shown in Figs 3 and 4.

### Eight-lane highway basic section

The traffic simulation parameters for an eight-lane highway basic section are listed in Table 2.

**Table 2:** Parameters for the eight-lane highway simulation.

Parameter	Value
Section length	18 km
Vehicle location sampling interval	15 s
Average traffic flow per lane	3,100 vph
Average speed	62 km/h
Average headway	1.16 s
One-way lanes	4
Minimum flow per lane	800 vph
Maximum flow per lane	4,200 vph

Based on the traffic simulation parameters in Table 2, when the average headway of the vehicles is 20 m, for a given road section, the average *CL* produced by periodic beacon messages is

$$\frac{8\left[lanes\right]*500m\left[com\_diameter\right]}{20m\left[between\;\_cars\right]}=200\left[cars/com\_range\right]$$

200[cars]*15pkts/s*800B/pkt*8bits/B=19.2Mbps

**Fig 5:**
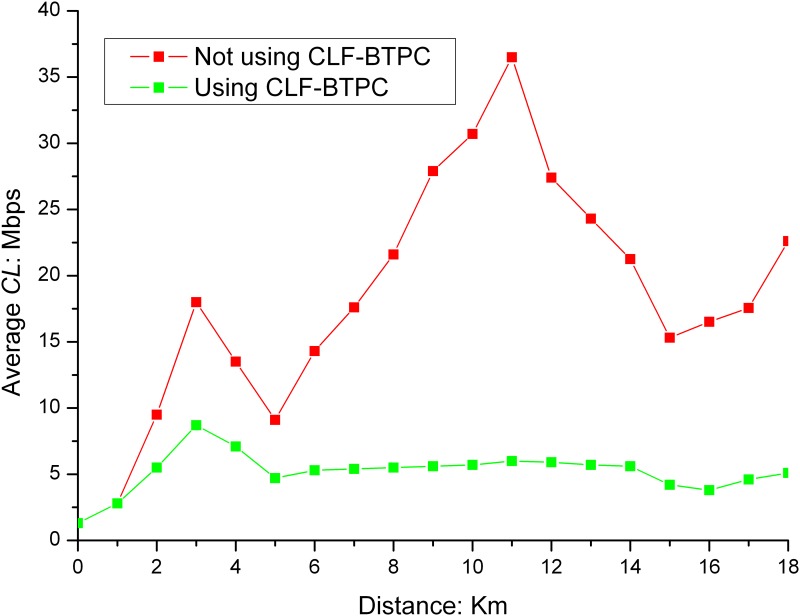
*CL* in different position on highway. As shown in Fig 5, after the CLF-BTPC algorithm is employed, the *CL* converged to a value within the predefined threshold range of 3–6 Mbps after a period of oscillation, which is determined by the distribution and fairness of the algorithm. When the run time of the algorithm is increased, the rate of convergence to the results increases.

**Fig 6:**
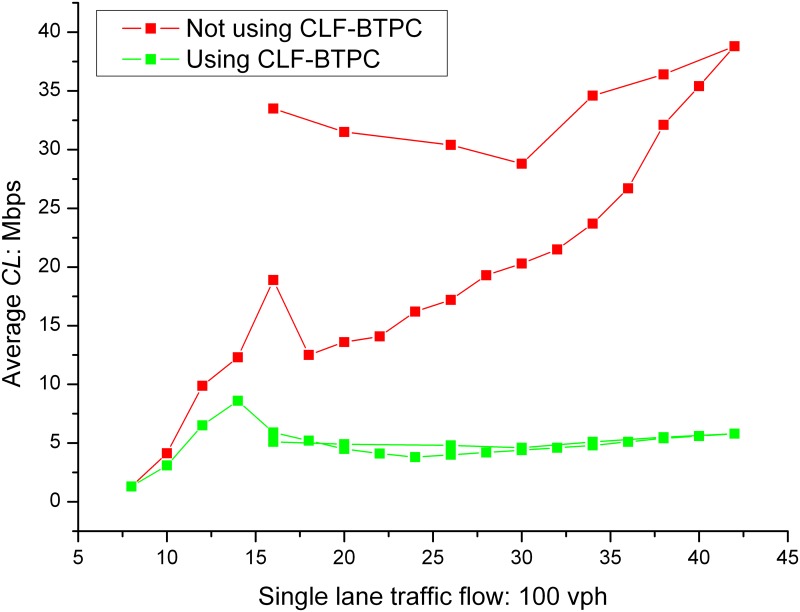
*CL* in different traffic flows in on highway. According to CLF-BTPC, as the beacon message transmission power changed, the *CSR* and number of shared nodes in a channel for each vehicle also changed; therefore, the wireless spatial multiplexing rate changed. When the transmission power changed by *kε*, the *CSR* was adjusted to (1±*kε*)·*CSR*_*ini*_, where *CSR*_*ini*_ is the initial *CSR*. The target node sends beacon messages at its highest transmission power, assuming that the *CL* fell within the predefined range. As shown in Fig 6, the *CL* is decreased to predefined threshold range of 3–6 Mbps after a period of oscillation. The simulation results show that CLF-BTPC algorithm sufficiently solves the *CL* congestion control problem in a VANET.

The average *CL*s before and after the CLF-BTPC algorithm was applied are shown in Figs 5 and 6.

The maximum and minimum power assignments *PA* can be calculated using the following equations:

$$PA_{\text{min}}=\frac{Min\_Adjust\_Load}{2*CSR_{\text{max}}*Vehicle\_Density*Load_{vehicle}}$$ (10)

$$PA_{\text{max}}=\frac{Max\_Adjust\_Load}{2*CSR_{\text{min}}*Vehicle\_Density*Load_{vehicle}}$$ (11)

In Eqs (10) and (11), *PA*_*min*_ is the minimum *PA*, *PA*_*max*_ is the maximum *PA* value, *CSR*_*max*_ is the maximum *CSR*, *CSR*_*min*_ is the minimum *CSR*, *Vehicle_Density* is the traffic flow density, *Load*_*vehicle*_ is the periodic beacon generation rate, *Min_Adjust_Load* is the minimum allowed *CL*, and *Max_Adjust_Load* is the maximum allowed *CL*.

Substituting the communication and traffic parameters in this paper into Eqs (10) and (11) yields the following minimum and maximum *PA* values:

$$PA_{\text{min}}=\frac{3Mbps}{2*1000m*1\left[cars/20m\right]*96\left[Kbps/car\right]}=0.31$$

$$PA_{\text{max}}=\frac{6Mbps}{2*500m*1\left[cars/20m\right]*96\left[Kbps/car\right]}=1.25$$

**Fig 7:**
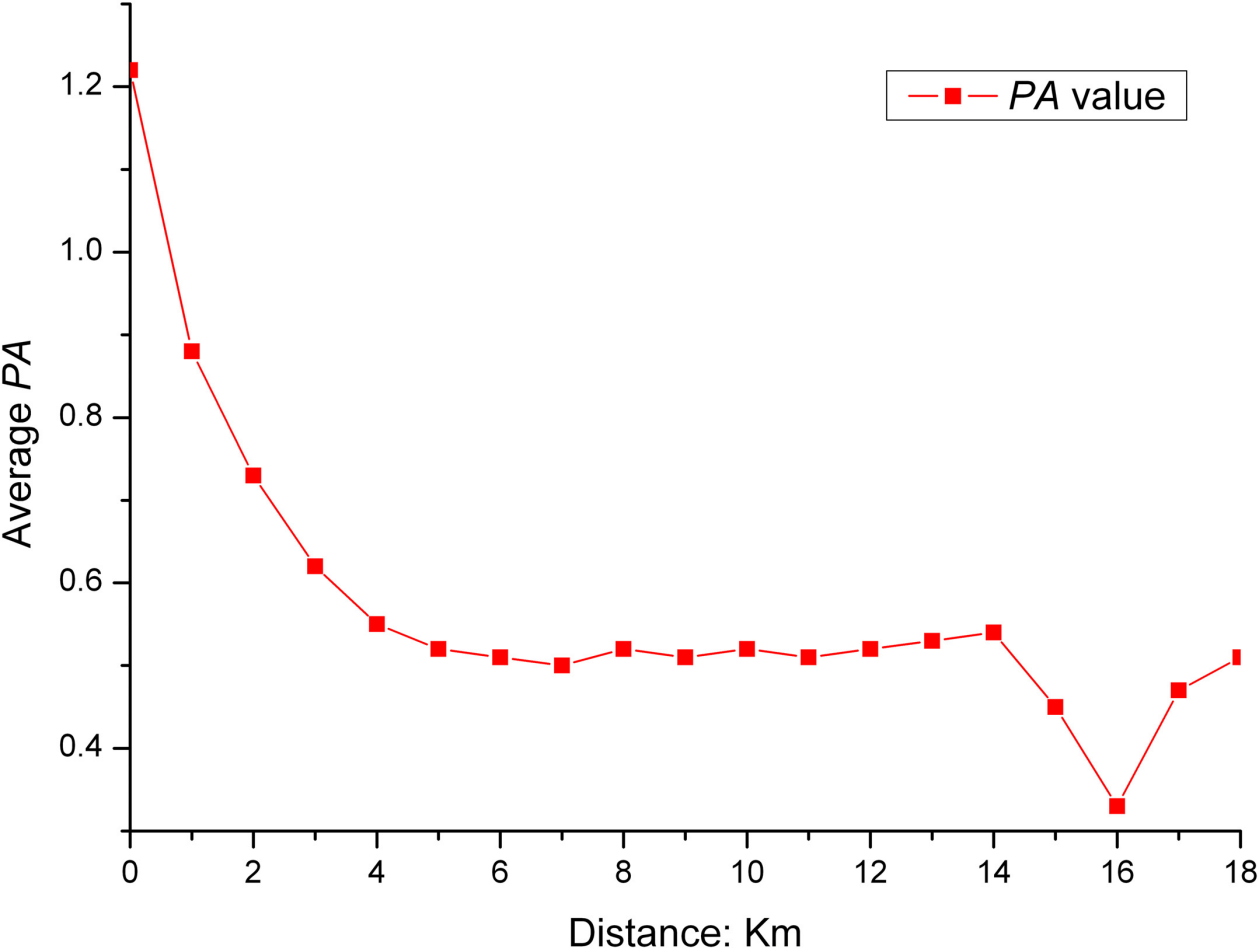
Relationship between *PA* and distance.

The *PA* is shown in Fig 7:

## Conclusion

In a VANET, designs for algorithm that control wireless channel loading under the premise of satisfactory vehicle safety are required. When the size and generation rate of periodic beacon messages have been reduced to minimum values, the transmission power of periodic beacon messages must be adjusted to effectively decrease the wireless channel load, prevent channel congestion, and guarantee maximal network connectivity. (1) In this study, the primary factors that influence channel load were selected, regression equations were constructed and Kalman filter-based recursion was performed. The actual road experimental results show that the KF-BCLF algorithm has a relatively high adaptability and forecasting accuracy. (2) According to a comparison of the channel load that was obtained from the KF-BCLF algorithm and the predefined maximum and minimum threshold values, the beacon transmission power is adjusted when the fairness is satisfactory. The simulation results show that the CLF-BTPC algorithm can respond to the dynamic topological structure of a mobile network, which enables beacon messages to be sent at the maximum transmission power and causes the channel load to rapidly converge to a value within a reasonable range.

The selection of additional parameters that influence a load may increase the accuracy of the KF-BCLF algorithm; however, the computational intensity and storage space requirements also increase. The impact of a wireless transmission environment was not included in the CLF-BTPC algorithm. For different channel fading conditions, the performance gain provided by controlling the transmission power generally produces different success rates for decoding beacon messages, which impacts the probability of successfully receiving a beacon message.

## Supporting Information

S1 Data There are two sheets (floating car and traffic simulation) in the file .
The sheet of floating car contains the traffic survey data collected by floating car, which is employed to verify the effectiveness of KF-BCLF. The sheet of traffic simulation contains the traffic simulation data from Paramics, which is employed to verify the effectiveness of CLF-BTPC.
(XLSX)
